# Supplementary material for: Redefining pleiotropic drug resistance in a pathogenic yeast: Pdr1 functions as a sensor of cellular stresses in Candida glabrata
Source: mSphere. 2023 Jun 26;8(4):e00254-23. doi: 10.1128/msphere.00254-23 (PMC10449514; doi:10.1128/msphere.00254-23)
Supplement: Figures S1 to S4 — Supplemental figures and legends. [file msphere.00254-23-s0001.pdf]

Supplemental Figure 1.

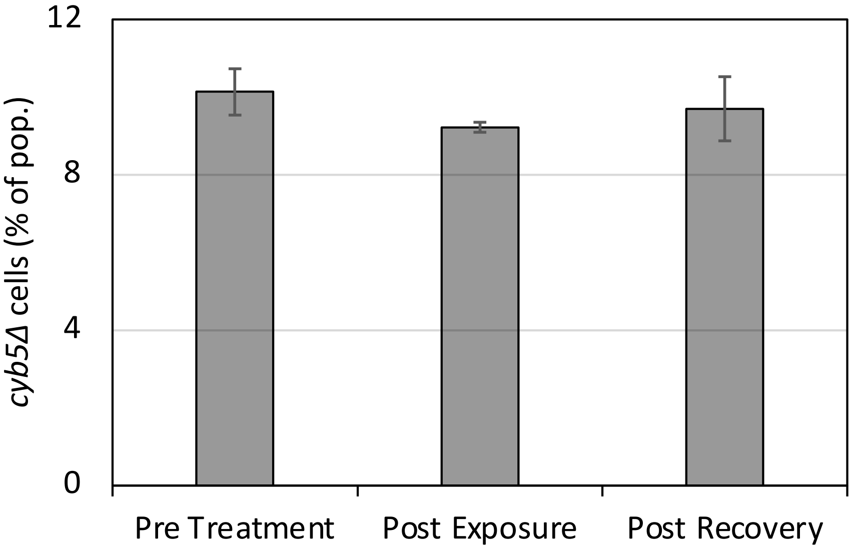

Supplemental Figure 1. Fluconazole sensitivity of *cyb5Δ* is rescued by co-culturing with BG14. Wild-type and *cyb5Δ* cells were grown to saturation and mixed at a 1:10 *cyb5Δ* to wild-type ratio. Mixtures were back diluted 1:100 into SCD media and exposed to 128 µg/mL of fluconazole for 24 hr. Cells were washed and resuspended in fresh SCD media for 24 hours. Relative abundance of wild-type to *cyb5Δ* mutants was calculated at the following time points, before drug exposure, post drug exposure and after recovery. Values are averaged from 3 biological replicates (±SD) .

Supplemental Figure 2

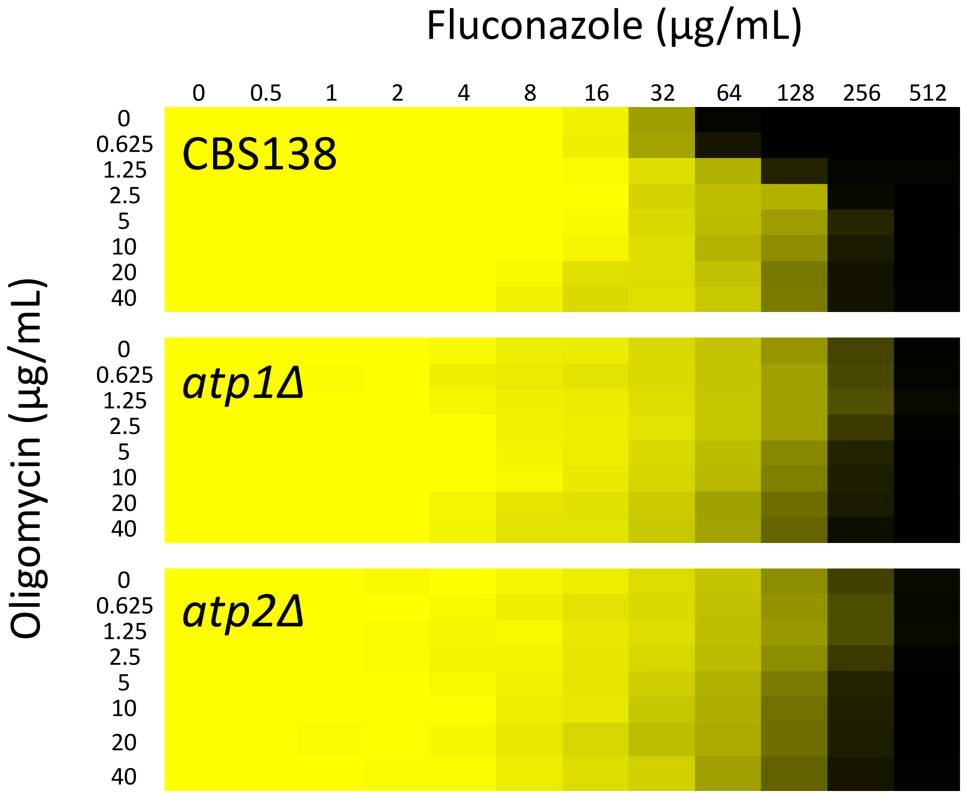

Supplemental Figure 2. Oligomycin antagonism of fluconazole effectiveness depends on ATP synthase. Growth of the wild-type CBS138, *atp1Δ,* and *atp2Δ* strains was measured at 600 nm after 20 hr incubation in SCD medium containing varying concentrations of fluconazole plus varying concentrations of oligomycin. Data are plotted as a heat map from saturation (yellow) to no growth (black). Strains were provided by David Perlin (Garcia-Rubio et al, mBio 12:e0195921, 2021)

Supplemental Figure 3

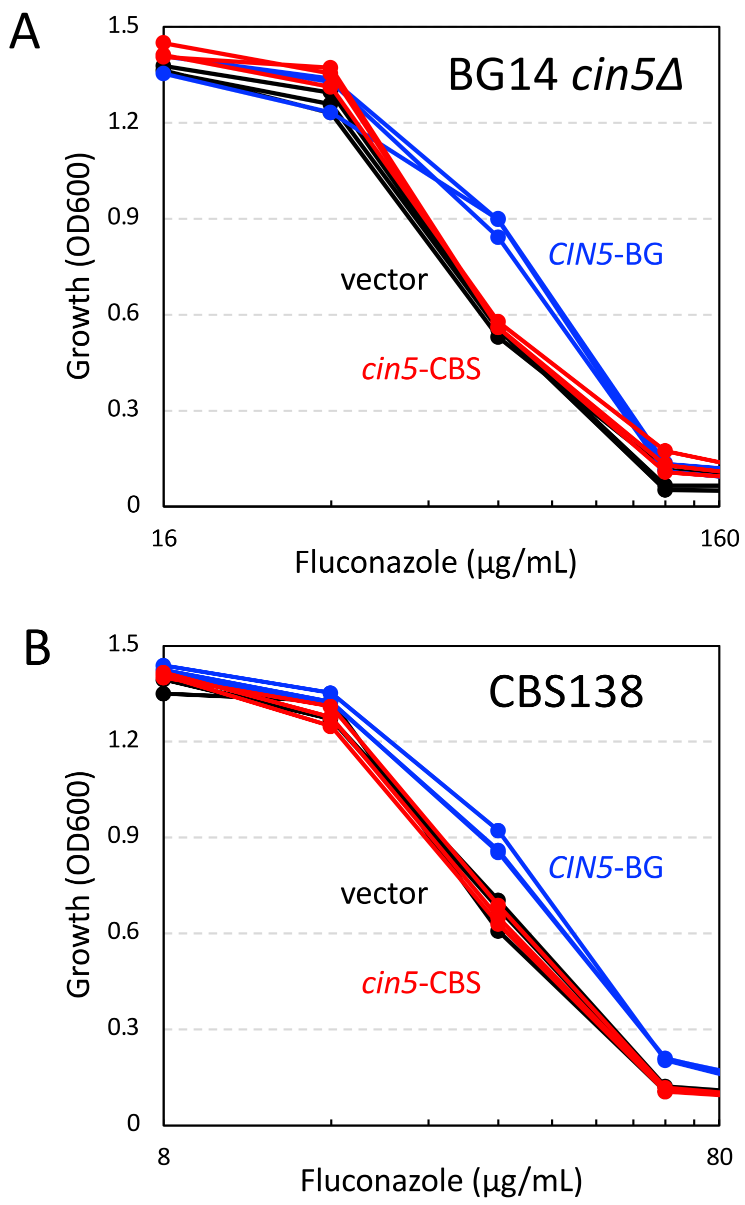

Supplemental Figure 3. An early stop codon in CBS138 inactivates *CIN5* function. Plasmids bearing *CIN5* from either BG2 or CBS138 parent strains were transformed into the BG14 *cin5Δ* (A) and CBS138 (B) strains and assayed in triplicate for susceptibility to fluconazole.

Supplemental Figure 4

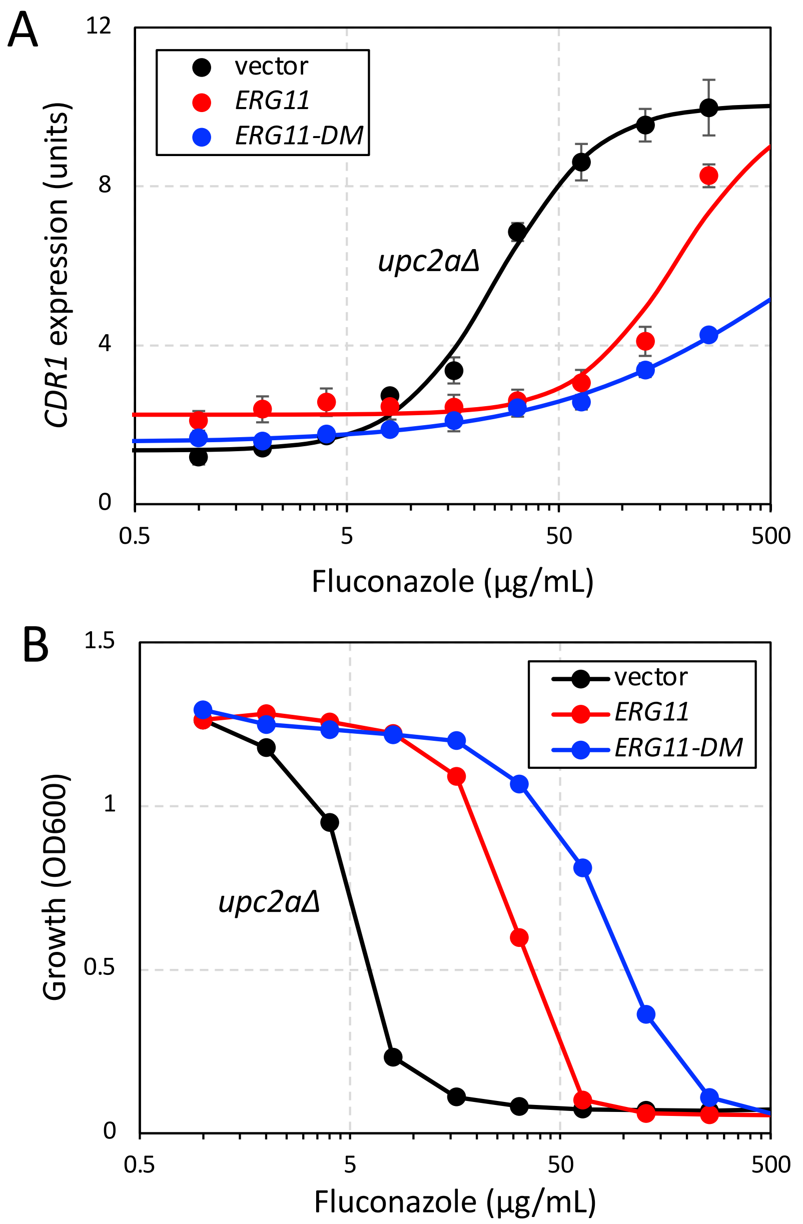

Supplemental Figure 4. Overexpressed Erg11 and Erg11-DM confer fluconazole resistance independent of Upc2A. The *upc2aΔ* mutant transformed with empty vector, *ERG11*, and *ERG11-DM* overexpression plasmids were grown in SCD-ura medium containing the indicated levels of fluconazole and analyzed for *CDR1* expression (A) and growth (B) as described in Fig. 6.
